# Evaluation of strategies to minimize ecotoxic side‐effects of sorbent‐based sediment remediation

**DOI:** 10.1002/jctb.5224

**Published:** 2017-03-06

**Authors:** Zhantao Han, Sebastian Abel, Jarkko Akkanen, David Werner

**Affiliations:** ^1^ Hebei and China Geological Survey Key Laboratory of Groundwater Remediation, Institute of Hydrogeology and Environmental Geology Chinese Academy of Geological Sciences China; ^2^ Department of Environmental and Biological Sciences University of Eastern Finland Finland; ^3^ School of Civil Engineering and Geosciences Newcastle University UK

**Keywords:** adsorption, char, environmental remediation, persistent organic pollutants (POPs), process optimization, pollution control

## Abstract

**BACKGROUND:**

In situ sorbent amendment for persistent organic pollutant sequestration in sediment has over the past 15 years steadily progressed from bench‐scale trials to full‐scale remediation applications. Hindering a wider technology uptake are, however, concerns about ecotoxic side‐effects of the most commonly used sorbent, activated carbon, on sensitive, sediment dwelling organisms like Lumbriculus variegatus. Using River Tyne sediment polluted with polycyclic aromatic hydrocarbons (PAHs) and L. variegatus as a case study, sorbent alternatives and magnetic sorbent‐recovery were investigated as potential engineering strategies to mitigate such ecotoxic side‐effects. The potential benefits of contacting the treated sediment with fresh River Tyne water, as would naturally occur over time in the intended applications, were studied.

**RESULTS:**

Magnetic biochar was identified as an effective PAH sorbent with less ecotoxic side‐effects than magnetic activated carbon. After 85.1–100% magnetic recovery of this biochar, no ecotoxic side‐effects on L. variegatus were measurable in the treated sediment. Results show that ecotoxic effects of magnetic activated carbon can be alleviated through sorbent recovery. In contrast, contacting treated sediment repeatedly with River Tyne water had no measurable benefits.

**CONCLUSIONS:**

Magnetic biochar is a promising sorbent material for the remediation of PAH polluted sediment. © 2017 The Authors. *Journal of Chemical Technology & Biotechnology* published by John Wiley & Sons Ltd on behalf of Society of Chemical Industry.

## INTRODUCTION

The benefits of hydrophobic organic contaminant (HOC) sequestration in sediment through the addition of a few percent by weight of activated carbon (AC) have been demonstrated in numerous laboratory^1^, ^2^, ^3^, ^4^, ^5^, ^6^ and field trials.^7^, ^8^, ^9^, ^10^, ^11^, ^12^, ^13^ AC amendment reduces HOC concentrations in sediment porewater,^14^ HOC uptake by sediment dwelling organisms,^15^ and HOC bioaccumulation in the aquatic food‐chain.^16^, ^17^ As demonstrated with field trials, AC‐based sediment remediation has become a technically feasible alternative to the dredging and off‐site disposal of contaminated sediments.^7^, ^13^, ^15^, ^18^

While organism survival in AC amended sediments is generally high, and several authors have reported only mild or no activated carbon amendment effects on biota,^19^, ^20^, ^21^ negative ecotoxic effects such as reduced wet tissue weights and lipid contents, reduced sediment feeding rates and AC amended sediment avoidance have also been reported in several organisms.^3^, ^22^, ^23^, ^24^ In particular, the oligochaete *Lumbriculus variegatus* was discovered to be a highly sensitive species negatively affected by AC.^22^ Doses as low as 0.25% dry sediment weight powdered activated carbon (PAC) were shown to reduce egestion rates, wet weights and lipid contents of this species.^23^ Uncertainty regarding the underlying causes of AC ecotoxicity in sediment has hindered mitigation efforts. Negative effects appear to be stronger in less nutritious sediments,^23^, ^25^ indicating that replenishing the nutrients removed by the AC adsorption may compensate for the unwanted side effects. Other authors submit that the AC directly causes ecotoxic effects.^22^ For example, morphological changes in the *L. variegatus* gut wall microvilli layer have been reported,^26^ motivating research into sorbent recovery methods.

Kupryianchyk *et al*.^27^ reported > 93% reductions in PCB and PAH sediment pore water concentrations for granular activated carbon sieved out of sediment, a positive effect on the survival of the waterlouse *Asellus aquaticus*, and no effect on the survival of *L. variegatus*, but no reduction in PCB mass fluxes from the sediment to overlaying water. For the use of magnetic activated carbon (MAC), we demonstrated a lasting 98% reduction in PAH sediment pore water concentrations before and after MAC recovery in laboratory experiments, but due to the relatively large, 8.1%, initial MAC dose used in that study and the nonlinearity of the ecotoxic response,^23^ a 1.8% dry sediment weight residual of unrecovered MAC still had strong negative effects on *L. variegates*.^28^

In the current study we therefore used a lower initial magnetic sorbent dose of 2.5% to sensitively test two complementary hypotheses about the best strategies for minimizing ecotoxic side‐effects of sorbent‐based sediment remediation: (i) effects can be minimized by using alternative sorbent materials; and (ii) effects can be minimized by effective sorbent recovery. Also, we investigated if re‐equilibrating the treated sediment with natural water to replenish contents bound by the sorbents can reduce ecotoxic effects.

## EXPERIMENTAL

### Sediment characterization

Superficial sediment was obtained from the intertidal zone of the River Tyne, at Gateshead, Newcastle upon Tyne, UK. The native organic carbon content was 3.0 ± 0.24%. The sediment polycyclic aromatic hydrocarbons (PAHs) were extracted by accelerated solvent extraction using hexane:acetone 50:50 v:v to determine total PAH concentrations by gas chromatography–mass spectrometry. All solvents used were pesticide residue grade, obtained from Sigma‐Aldrich, St. Louis, USA. Available PAH concentrations were determined by using polyethylene (PE) passive samplers.^29^ More detailed method descriptions are available in previous publications.^28^, ^30^

### Preparation of magnetic activated carbon and biochar

A high quality AC produced from anthracite coal (75–300 µm type TOG) for point‐of‐use drinking water treatment, also used in previous sediment amendment studies,^1^ was obtained from Calgon (Pittsburgh, United States). A wood‐based biochar, which is a strong, well‐characterized PAH adsorbent,^31^ was obtained from Romchar (Harghita, Romania). These sorbents were labeled TOG, and Bio, respectively. Bio was ground with a ceramic mortar to achieve a size distribution < 64 µm, the size of PAC. TOG and Bio were magnetized as detailed in a previous study.^31^ The magnetic carbon materials were labeled MagTOG and MagBio, respectively.

### Sediment remediation with magnetic activated carbon or biochar amendment

Eight aliquots of 283.7 g (120.0 g dry weight) River Tyne sediment were put into wide mouth amber glass jars, and 3.00 g MagTOG or MagBio (w/w, equal to 2.5% MAC or MBC content, or 1.6% AC or BC content) were added into three of the jars, respectively. The two remaining jars without magnetic carbon amendment were used as controls. Three pre‐cleaned PE samplers were put into each jar, and the bottles were sealed with PTFE lined caps and shaken horizontally at 100 rpm. After 3 months, all the PE samplers were recovered, wiped cleaned, and extracted twice for 24 h with 10 mL hexane:acetone 80:20 v:v. The combined extracts were analyzed by GC‐MS as described in Han *et al*.^28^ From two of the three amended samples, MagTOG and MagBio were recovered using a magnetic rod (Eclipse Magnetics, Sheffield, S4‐7QQ, UK), and cleaned from comingled sediment by swirling the rod in deionized water, and oven‐dried to determine MAC recovery. Naturally present magnetic minerals in control samples were also recovered by the same method to accurately calculate the MAC recovery. One of the two MagTOG or MagBio recovered sediment samples, and one of the control sediment samples, were contacted with fresh River Tyne water to investigate an eventual benefit of re‐equilibration as would naturally occur over time in the intended application. Glass beakers containing the treated sediment (∼283.7 g) were filled to the 500 mL mark with River Tyne water, stirred to suspend the sediment, and allowed to settle overnight, before the supernatant was decanted. This procedure was repeated five times. The sediment samples were labelled Control, Control‐RW, MagTOG, MagTOG‐R, MagTOG‐RW, MagBio, MagBio‐R, MagBio‐RW, respectively, where 'R' indicates that magnetic materials were recovered, and 'RW' indicates the sediment was also contacted with River Tyne water following sorbent recovery.

The iron content of the recovered magnetic materials was measured by digesting 0.5 g of MagTOG or MagBio or 0.36 g of recovered sediment magnetic material in 90 mL of deionized water with 6 mL of concentrated nitric acid and 6 mL of concentrated hydrochloric acid, sonicating for 20 min and then shaking for 5 days. The digestates were diluted 1:3 with distilled water for ICP‐OES analysis on a Varian Vista MPX axial ICP‐OES with CCD, operated according to standard methods for examination of water and wastewater.

### Ecotoxicity tests

Control, Control‐RW, MagTOG, MagTOG‐R, MagTOG‐RW, MagBio, MagBio‐R, and MagBio‐RW sediment samples were used in ecotoxicity tests with *L. variegatus*. Quadruplicate microcosms were set up in 200 mL glass jars filled with 50 g (wet weight) of sediments and 130 mL of overlying artificial freshwater. The starting mass of *L. Variegatus* was 6.3 ± 1.6 mg fresh weight per worm, the initial dry weight was 17.7% of the fresh weight, and 10 worms were added to each microcosm. The worm dry weight to sediment organic carbon ratio was < 2%. Wet biomass weight, dry biomass weight and reproduction of *L. variegatus* were used as sensitive endpoints to examine the ecological side‐effects of treatments. A detail method description can be found in Nybom *et al*.^23^

## RESULTS AND DISCUSSION

### PAH concentrations in sediment and PE samplers

**Figure 1:**
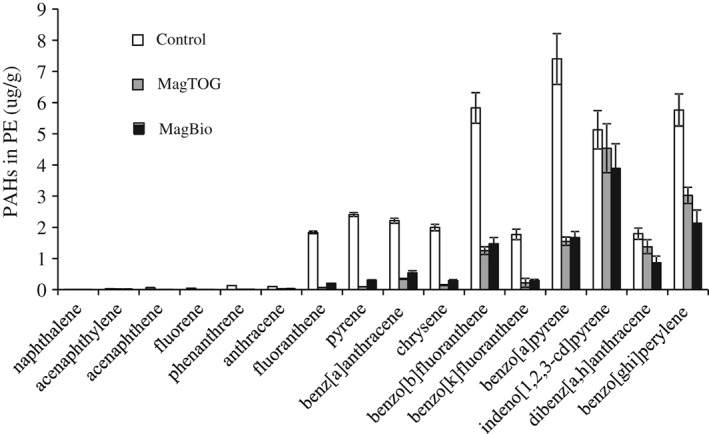
PAH concentrations in the PE samplers in River Tyne sediment amended with different magnetic sorbent materials (MagTOG, MagBio) compared with the unamended sediment (Control).

The solid phase PAH concentration in the River Tyne sediment was 6.05 ± 0.42 µg g^−1^ for the 16 US EPA PAHs, which was lower than in the sediment collected from the same location in March 2013 for a previous study (16.08 ± 0.60 µg g^−1^),^28^ but sufficient to quantify treatment benefits: After 3 months, available PAH concentrations in PE samplers of the unamended, MagTOG amended and MagBio amended River Tyne sediment batches were 36.5 ± 10.3, 12.7 ± 1.7 and 11.7 ± 2.0 µg g^−1^, respectively (Fig. 1), equivalent to a 65.3% and 67.9% reduction in the PAH availability for MagTOG and MagBio amended in comparison with the unamended sediment. While the magnetic activated carbon tended to perform slightly better for smaller molecular weight PAHs, the magnetic biochar performed slightly better for larger PAHs (Fig. 1), which may be explained by distinct molecular sieving effects of the two sorbents. For comparison, a 74% PAH availability reduction for the 16 US EPA PAHs in a harbor sediment after one month contact was reported by Zimmerman *et al*. for a 3.4% non‐magnetic TOG dose,^32^ which, due to the lower carbon content of magnetite impregnated sorbents, would correspond to an equivalent 5.5% MagTOG dose. Important for the purpose of this study is that in terms of overall treatment effectiveness (percentage reduction for the sum of PAHs) the two magnetic sorbent types were statistically indistinguishable (t‐test, two‐tailed, *P* = 0.50).

### Magnetic activated carbon and biochar recovery

After 3 months of sediment amendment, the recovery rate of MagTOG was only 25.9–36.9%, while the recovery rate of MagBio was 85.1–100% (Table 1). The difference in particle sizes between MagTOG and MagBio probably explain these different recoveries, since the iron content of the recovered particles was lowest for the MagBio, which nonetheless had the highest recovery. The particle size of MagTOG was 75–300 µm, while the particle size of MagBio was < 64 µm. Larger particles are more difficult to neatly separate from commingled sediment attached to the magnetic rod because of their heavier weight. The iron element content of the recovered MagTOG was not significantly different from the original MagTOG (t‐test, two‐tailed, P = 0.26), but the recovered MagBio had significantly lower elemental iron content than the original MagBio (t‐test, two‐tailed, P = 0.03), suggesting partial dissolution of the magnetite particle impregnation in the sediment. The substantial reduction in elemental iron content of recovered MagBio particles, and perhaps also the unrecovered MagTOG particles, might result from the anaerobic conditions in the fine‐grained River Tyne sediment, but exact mechanisms need to be confirmed in a follow‐on study. For the purpose of this study, the main consideration is that MagTOG residuals in sediment following the magnetic recovery are much higher than MagBio residuals (1.6–1.9% versus 0–0.4% dry sediment weight, respectively).

**Table 1:** Sample information for the ecotoxicity tests, recovery and iron content measurements

ID	Magnetic sorbent added (g)	Magnetic sorbent recovered (g)	Magnetic sorbent recovery (%)	Magnetic sorbent dose (% dw)	Iron content of sorbents (mg g^−1^)
Control	0	NA	NA	NA	NA^a^
b Control‐RW	0	NA	NA	NA	NA
MagTOG	3	NA	NA	2.5	271 ± 31^c^
b MagTOG‐R	3	1.11	36.9	1.58	294 ± 17
b MagTOG‐RW	3	0.78	25.9	1.85	349
MagBio	3	NA	NA	2.5	266 ± 57^c^
b MagBio‐R	3	2.56	85.1	0.37	109 ± 8.0
b MagBio‐RW	3	3	100	0	154

a not available

b The magnetic sorbents in MagTOG‐R and MagBio‐R were recovered after 3 months by magnetic separation; the magnetic sorbents in MagTOG‐RW and MagBio‐RW were also recovered, and the sediment was then contacted with fresh River Tyne water as described in the method section.

c Iron content of the original magnetic sorbent.

### Ecotoxic effects of sediment remediation with magnetic activated carbon or biochar

**Figure 2:**
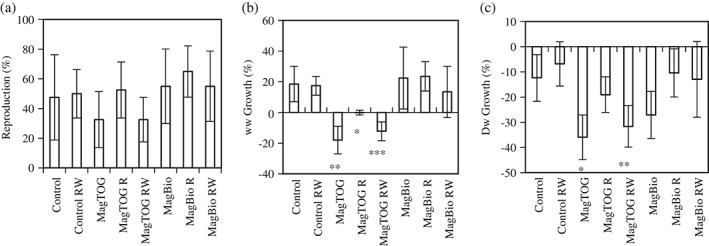
Lumbriculus variegatus reproduction (a), wet weight growth (b) and dry weight growth (c), in differently treated sediments over 28 days. Significant differences relative to the respective control are indicated by ‘*’ (t‐test, two‐tailed, P < 0.05), ‘**’ (t‐test, two‐tailed, P < 0.01), ‘***’ (t‐test, two‐tailed, P < 0.001).

Lumbriculus variegatus reproduction was not measurably affected by magnetic sorbent amendments in any of the treatments (Fig. 2(a), t‐test, two‐tailed, all P > 0.16). The wet weight (ww) growth of L. variegatus was, on the other hand, significantly inhibited by an amendment of 2.5% MagTOG, and also by the 1.6–1.9% unrecovered MagTOG residuals (Fig. 2(b), t‐test, two‐tailed, P =0.003, 0.02 and 0.0005, respectively). MagBio treatments had no statistically significant effects on L. variegatus ww growth (Fig. 2(b), t‐test, two‐tailed, all P > 0.52). The dry weight (dw) growth of L. variegatus was significantly inhibited by MagTOG and MagTOG‐RW treatments (Fig. 2(c), t‐test, two‐tailed, P =0.01 and 0.006, respectively). The negative dw growth is probably due to a reduction in L. variegatus storage lipid contents often observed in ecotoxicity tests with natural sediments.^22^ There were no statistically significant effects of MagBio treatments on L. variegatus dw growth (Fig. 2(c)), although the 2.5% MagBio amendment result was fairly close to the level of significance (t‐test, two‐tailed, P = 0.09).

The effect of the 2.5% MagTOG amendment observed in this study, although significant, was mild compared with the unrecovered 1.8% MagCoalAC residual in a previous study with sediment from the same location (−82% reproduction and −65% wet weight growth),^28^ probably because of the greater TOG particle‐size (75–300 µm for MagTOG vs 1–50 µm for MagCoalAC).^23^ The particle size of MagTOG used in this study is mostly greater than the ingestible particle range of *L. variegates*.^23^ On the other hand, the smaller effect on *L. variegatus* ww of the 2.5% MagBio amendment, all of it in the ingestible particle range (<64 µm), compared with the 2.5% MagTOG amendment, shows that the biochar matrix has lesser ecotoxicity than the activated carbon matrix (*t*‐test, two‐tailed, *P* = 0.015), since based on the particle size only, one would have anticipated greater ecotoxicity for the finer sized sorbent material. In summary, these observations demonstrate significant scope for optimizing the choice of the sorbent amendment material, and its particle size, through combined consideration of treatment effectiveness (Fig. 1) and ecotoxic side‐effects (Fig. 2).

Following the 85.1–100% magnetic recovery of MagBio, all of the three ecotoxicity assessments (reproduction, ww and dw growth of *L. variegatus*) in MagBio‐R were statistically indistinguishable from the control (Fig. 2, *t*‐test, two‐tailed, all *P* > 0.33). The 36.9% recovery of MagTOG significantly alleviated the inhibition of ww growth and dw growth of *L. variegatus* by 2.5% MagTOG (*t*‐test, two‐tailed, *P* = 0.008 and 0.03, respectively). There was also an apparent, although not statistically significant, benefit of the MagBio‐R recovery on the dw growth of *L. variegatus* (*t*‐test, two‐tailed, *P* = 0.07). These observations demonstrate the benefits of sorbent recovery. It would appear that the sorbent amendment itself, rather than the binding of nutritious content from sediment, caused ecotoxic effects in this study, since adsorbed sediment components would have been removed together with the recovered sorbents, leaving behind a depleted sediment, which would still cause ecotoxic effects. However, nutrient availability was not directly assessed in this study. With the methods employed it can also not be excluded that the observed reduced growth effects occur due to the binding of nutritious contents by the sorbents during sediment digestion, i.e. happening within the gut of the worms, rather than externally. Also, repeated contact of the sediment with large volumes of River Tyne water did not alleviate ecotoxic effects in any of the treatments, which indicates that re‐equilibration with natural water cannot rapidly compensate for the unwanted side‐effects of the sorbent amendment. For MagTOG, the recovery rate from the MagTOG‐RW batch was lower (25.9%) than that of the MagTOG‐R batch (36.9%), and this may explain an apparent adverse ecotoxic effect of the subsequent contact with River Tyne water in this treatment.

### Outlook

Compromises can be found between maximizing the treatment benefits of sorbent amendments and minimizing unwanted ecotoxic side‐effects: reducing the magnetic sorbent dose from 8.1% in the previous study^28^ to 2.5% in the current study, increasing the magnetic AC particle size to just above the ingestible limit of *L. variegatus*, replacing AC with biochar as the carbonaceous sorbent matrix, and the recovery of magnetic sorbents, all these measures, to a variable extent, alleviated or annulled ecotoxic amendment effects to the sediment worm *L. variegatus*, which had sensitively responded to even low AC amendment doses in earlier studies with clean^23^ and contaminated sediments.^26^, ^28^ MagBio appears to present a particularly good candidate material for sediment remediation, considering, in addition to the demonstrated remediation effectiveness, lower ecotoxicity and good magnetic recoverability. Also, biochar is produced from renewable biomass and has lower net environmental impacts compared with fossil coal‐derived AC in sediment remediation applications.^33^ The low ecotoxicity of biochar suggests it could be used as amendment to sediments without magnetization and recovery, perhaps at a slightly higher dose to further improve the observed 67.9% reduction in the bioavailability of PAHs. The biochar magnetization method is useful in situations, where leaving the sorbent and associated pollutants in the sediment is a major stakeholder concern. In light of these encouraging findings, further work should identify the factors determining the magnetic sorbent stability and recoverability from sediment, as our iron quantification results suggest that the magnetite impregnation of the particles may partially dissolve in prolonged contact with sediment, and the larger‐scale feasibility of the magnetic recovery process also needs to be demonstrated.

## References

[1] Zimmerman JR , Ghosh U , Millward RN , Bridges TS and Luthy RG , Addition of carbon sorbents to Reduce PCB and PAH bioavailability in marine sediments: physicochemical tests. Environ Sci Technol 38:5458–5464 (2004). 15543751 10.1021/es034992v

[2] Werner D , Higgins CP and Luthy RG , The sequestration of PCBs in Lake Hartwell sediment with activated carbon. Water Res 39:2105–2113 (2005). 15922398 10.1016/j.watres.2005.03.019

[3] Millward RN , Bridges TS , Ghosh U , Zimmerman JR and Luthy RG , Addition of activated carbon to sediments to reduce PCB bioaccumulation by a polychaete (*Neanthes arenaceodentata*) and an amphipod (*Leptocheirus plumulosus*). Environ Sci Technol 39:2880–2887 (2005). 15884389 10.1021/es048768x

[4] Sun X and Ghosh U , The effect of activated carbon on partitioning, desorption, and biouptake of native polychlorinated biphenyls in four freshwater sediments. Environ Toxicol Chem 27:2287–2295 (2008). 18517308 10.1897/08-020.1

[5] Lin D , Cho YM , Werner D and Luthy RG , Bioturbation delays attenuation of DDT by clean sediment cap but promotes sequestration by thin‐layered activated carbon. Environ Sci Technol 48:1175–1183 (2014). 24359108 10.1021/es404108h

[6] Choi YJ , Cho YM , Werner D and Luthy RG , *In situ* sequestration of hydrophobic organic contaminants in sediments under stagnant contact with activated carbon. 2. Mass transfer modeling. Environ Sci Technol 48:1843–1850 (2014). 24410479 10.1021/es404209v

[7] Cho YM , Ghosh U , Kennedy AJ , Grossman A , Ray G , Tomaszewski JE *et al*, Field application of activated carbon amendment for *in situ* stabilization of polychlorinated biphenyls in marine sediment. Environ Sci Technol 43:3815–3823 (2009). 19544893 10.1021/es802931c

[8] Cornelissen G , Krusa ME , Breedveld GD , Eek E , Oen AMP , Arp HPH *et al*, Stokland O and Gunnarsson JS , Remediation of contaminated marine sediment using thin‐layer capping with activated carbon – a field experiment in Trondheim Harbor, Norway. Environ Sci Technol 45:6110–6116 (2011). 21671651 10.1021/es2011397

[9] Oen AMP , Janssen EML , Cornelissen G , Breedveld GD , Eek E and Luthy RG , *In situ* Measurement of PCB pore water concentration profiles in activated carbon‐amended sediment using passive samplers. Environ Sci Technol 45:4053–4059 (2011). 21473574 10.1021/es200174v

[10] Beckingham B and Ghosh U , Field‐scale reduction of PCB bioavailability with activated carbon amendment to river sediments. Environ Sci Technol 45:10567–10574 (2011). 22077959 10.1021/es202218p

[11] Cho Y‐M , Werner D , Choi Y and Luthy RG , Long‐term monitoring and modeling of the mass transfer of polychlorinated biphenyls in sediment following pilot‐scale *in situ* amendment with activated carbon. J Contam Hydrol 129–130:25–37 (2012). 10.1016/j.jconhyd.2011.09.009 22055155

[12] Kupryianchyk D , Peeters E , Rakowska MI , Reichman EP , Grotenhuis JTC and Koelmans AA , Long‐term recovery of benthic communities in sediments amended with activated carbon. Environ Sci Technol 46:10735–10742 (2012). 22934596 10.1021/es302285h

[13] Cornelissen G , Amstaetter K , Hauge A , Schaanning M , Beylich B , Gunnarsson JS *et al*, Large‐scale field study on thin‐layer capping of marine PCDD/F‐contaminated sediments in Grenlandfjords, Norway: physicochemical effects. Environ Sci Technol 46:12030–12037 (2012). 23046183 10.1021/es302431u

[14] Werner D , Ghosh U and Luthy RG , Modeling polychlorinated biphenyl mass transfer after amendment of contaminated sediment with activated carbon. Environ Sci Technol 40:4211–4218 (2006). 16856737 10.1021/es052215k

[15] Ghosh U , Luthy RG , Cornelissen G , Werner D and Menzie CA , *In situ* sorbent amendments: a new direction in contaminated sediment management. Environ Sci Technol 45:1163–1168 (2011). 21247210 10.1021/es102694h PMC3037809

[16] Kupryianchyk D , Rakowska MI , Roessink I , Reichman EP , Grotenhuis JTC and Koelmans AA , *In situ* treatment with activated carbon reduces bioaccumulation in aquatic food chains. Environ Sci Technol 47:4563–4571 (2013). 23544454 10.1021/es305265x

[17] Fadaei H , Watson A , Place A , Connolly J and Ghosh U , Effect of PCB bioavailability changes in sediments on bioaccumulation in fish. Environ Sci Technol 49:12405–12413 (2015). 26402889 10.1021/acs.est.5b03107

[18] Patmont CR , Ghosh U , LaRosa P , Menzie CA , Luthy RG , Greenberg MS *et al*, *In situ* sediment treatment using activated carbon: a demonstrated sediment cleanup technology. Integrated Environ Assess Manage 11:195–207 (2015). 10.1002/ieam.1589 PMC4409844 25323491

[19] Janssen EML , Croteau M‐N , Luoma SN and Luthy RG , Measurement and modeling of polychlorinated biphenyl bioaccumulation from sediment for the marine polychaete *Neanthes arenaceodentata* and response to sorbent amendment. Environ Sci Technol 44:2857–2863 (2010). 20384377 10.1021/es901632e

[20] Kupryianchyk D , Reichman EP , Rakowska MI , Peeters ETHM , Grotenhuis JTC and Koelmans AA , Ecotoxicological effects of activated carbon amendments on macroinvertebrates in nonpolluted and polluted sediments. Environ Sci Technol 45:8567–8574 (2011). 21846106 10.1021/es2014538

[21] Beckingham B , Buys D , Vandewalker H and Ghosh U , Observations of limited secondary effects to benthic invertebrates and macrophytes with activated carbon amendment in river sediments. Environ Toxicol Chem 32:1504–1515 (2013). 23554105 10.1002/etc.2231

[22] Jonker MTO , Suijkerbuijk MPW , Schmitt H and Sinnige TL , Ecotoxicological effects of activated carbon addition to sediments. Environ Sci Technol 43:5959–5966 (2009). 19731704 10.1021/es900541p

[23] Nybom I , Werner D , Leppänen MT , Siavalas G , Christanis K , Karapanagioti HK *et al*, Responses of *Lumbriculus variegatus* to activated carbon amendments in uncontaminated sediments. Environ Sci Technol 46:12895–12903 (2012). 23153215 10.1021/es303430j

[24] Janssen EML and Beckingham BA , Biological responses to activated carbon amendments in sediment remediation. Environ Sci Technol 47:7595–7607 (2013). 23745511 10.1021/es401142e

[25] Janssen EML , Choi Y and Luthy RG , Assessment of nontoxic, secondary effects of sorbent amendment to sediments on the deposit‐feeding organism *Neanthes arenaceodentata* . Environ Sci Technol 46:4134–4141 (2012). 22372688 10.1021/es204066g

[26] Nybom I , Waissi‐Leinonen G , Maenpaa K , Leppanen MT , Kukkonen JVK , Werner D *et al*, Effects of activated carbon ageing in three PCB contaminated sediments: sorption efficiency and secondary effects on *Lumbriculus variegatus* . Water Res 85:413–421 (2015). 26364225 10.1016/j.watres.2015.08.044

[27] Kupryianchyk D , Noori A , Rakowska MI , Grotenhuis JTC and Koelmans AA , Bioturbation and dissolved organic matter enhance contaminant fluxes from sediment treated with powdered and granular activated carbon. Environ Sci Technol 47:5092–5100 (2013). 23590290 10.1021/es3040297

[28] Han Z , Sani B , Akkanen J , Abel S , Nybom I , Karapanagioti HK *et al*, A critical evaluation of magnetic activated carbon's potential for the remediation of sediment impacted by polycyclic aromatic hydrocarbons. J Hazard Mater 286:41–47 (2015). 25550081 10.1016/j.jhazmat.2014.12.030

[29] Hale SE and Werner D , Modeling the mass transfer of hydrophobic organic pollutants in briefly and continuously mixed sediment after amendment with activated carbon. Environ Sci Technol 44:3381–3387 (2010). 20392086 10.1021/es903582n

[30] Hale S , Martin T , Goss KU , Arp H and Werner D , Partitioning of organochlorine pesticides from water to polyethylene passive samplers. Environ Pollut 158:2511–2517 (2010). 20398988 10.1016/j.envpol.2010.03.010

[31] Han Z , Sani B , Mrozik W , Obst M , Beckingham B , Karapanagioti HK *et al*, Magnetite impregnation effects on the sorbent properties of activated carbons and biochars. Water Res 70:394–403 (2015). 25555224 10.1016/j.watres.2014.12.016

[32] Zimmerman JR , Werner D , Ghosh U , Millward RN , Bridges TS and Luthy RG , Effects of dose and particle size on activated carbon treatment to sequester polychlorinated biphenyls and polycyclic aromatic hydrocarbons in marine sediments. Environ Toxicol Chem 24:1594–1601 (2005). 16050574 10.1897/04-368r.1

[33] Sparrevik M , Saloranta T , Cornelissen G , Eek E , Fet AM , Breedveld GD *et al*, Use of life cycle assessments to evaluate the environmental footprint of contaminated sediment remediation. Environ Sci Technol 45:4235–4241 (2011). 21520943 10.1021/es103925u

